# Prevalence of Chronic Complication among Type 2 Diabetics Attending Primary Health Care Centers of Al Ahsa District of Saudi Arabia: A Cross Sectional Survey

**DOI:** 10.5539/gjhs.v6n4p245

**Published:** 2014-04-21

**Authors:** Ataur Rahman Khan, Zaki Naser Al-Abdul Lateef, Shaheen Fatima, Sana Abdullah Ahmad Al Yousuf, Syed Zakaullah Khan Afghan, Salah Al Marghani

**Affiliations:** 1 Al Omran Primary Health Care Centre, Al Ahsa, Saudi Arabia; 2 Primary Health Care, Health Directorate, Al Ahsa District, Saudi Arabia; 3 University of Windsor Medical School, St. Kitts, Saudi Arabia; 4 CBR Co Ordinator in Public Health, Eastern Province, Saudi Arabia; 5 Primary Health Care Center, Saudi Arabia; 6 Ministry of Health, Al Hofuf, Saudi Arabia

**Keywords:** type 2 diabetes, Hb1AC, chronic complications, hypertension

## Abstract

**Background:**

The morbidity and mortality related to diabetes is a great global concern. The knowledge of chronic complications of diabetes and associated co morbidity factors is very important for formulating the necessary policies and action plan.

**Aims:**

To determine the prevalence of chronic complications and comorbidity among the type 2 diabetics attending the primary health care centers of Al Ahsa district of Saudi Arabia.

**Material and Methods:**

This cross sectional retrospective survey was carried out on 506 type 2 diabetic patients attending the different primary health care centers of ministry of health, Al Ahsa. Data regarding the co morbidity factors and chronic complications were recorded from the health records of the selected diabetic patients. Data analysis was done by SPSS version 16. A p<0.05 was considered significant for all statistical calculations.

**Results:**

Overall 72.72% (95% CI 69.78-74.45) of the study subjects were suffering from one or more complications of diabetic mellitus. Among them 33.39% (165) were suffering from single, 25.29% (128) with two and 15% (75) from more than two complications. The overall prevalence of complication among the female subjects was significantly higher than the male (78.16%, 95% CI 76.76-84.40 Vs 65.76%, 95% CI 61.63-69.89, p=.038). The chronic complication was higher among the urban population than the rural population (77.3% 95% CI 72.88-80.26 Vs 69.78% 95% CI 66.1%-76.92%, p=.035).

**Conclusion:**

The result showed a high percentage of chronic complications among the diabetic patients of this region. The high percentage of obesity, hypertension and dyslipidaemia among them are important co morbidity factors which if not controlled can cause further increase in the number of chronic complications.

## 1. Introduction

Diabetes Mellitus is a global chronic public health problem. The magnitude of this problem is expected to rise enormously with the predicted increase in its prevalence in the coming years. According to one estimate the number of adults suffering from diabetes is likely to more than double by 2025 from its existing number of 177 million (Sierra, 2009). The morbidity and mortality related to diabetes is a great global concern. The data from various studies has found diabetes as the growing cause of disability and premature death mainly through its chronic complications. Studies conducted in various countries have shown increased incidence of micro as well as the macro vascular complications among the diabetic patients. Diabetes mellitus is one of the major risk factors for cardio vascular diseases. Approximately 30% of patients treated in cardiovascular intensive care units have diabetes (Kengne & Mbanya, 2005). One study conducted in the central province of Saudi Arabia has found 24.1% of the diabetic patient suffering from cardiac ischemic disease. Diabetic retinopathy is fast emerging as the one of the commonest causes of blindness. According to WHO Diabetic retinopathy is the commonest cause of blindness among the working age group of the developed nation and over 2.5 million people are blind globally due to diabetes (Khan, Wiseberg, Lateef, & Khan, 2010). Recent studies conducted in central and eastern provinces of Saudi Arabia have found 34 and 31% of diabetic retinopathy among the diabetic patients (Alwakeel, Al-Suwaida, Isnani, Al-Harbi, & Alam, 2009; Khan et al., 2010). Diabetic nephropathy is similarly a serious complication of diabetes and affects 30-40% of all patients with diabetes (Caring for Diabetes.com, 2012). In a clinic based study in Riyadh and a primary health care based study in Abha (Saudi Arabia), the researchers have found 41.3% and 12.8% of the diabetic patients suffering from frank proteinuria (Farag & Al Wakeel, 2011; Al-Homrany & Abdelmoneim, 2004).

The incidence of cerebrovascular infarction has been found to be 2.5- to 3.5 fold greater in the diabetic population aged 45 to 74 years than in the non diabetic population (Currie, Morgan, Gill, Stott, & Peters, 1997). In a hospital study conducted in Riyadh researchers have found a prevalence of 17.6% of stroke among the diabetics attending the hospital while in a population based study in UAE a prevalence of 3,5% (95% CI:1.9-5.1) of cerebrovascular complication was documented (Alwakeel et al., 2009; Al-Maskari, El-Sadig, & Norman, 2007).

The morbidity caused by diabetic foot complication is one of the most expensive diabetes (DM) complications to treat. According to one study conducted in Saudi Arabia, the prevalence of diabetic foot was 6% among the diabetic patients and 1.5 % had amputed foot (Abougalambou, Hassali, Sulaiman, & Abougalambou, 2011).

## 2. Materials & Methods

The local directorate of Ministry of Health has granted permission to pursue this study and data were extracted from the patient’s records. Despite the fact that there was no direct involvement of the study subjects in this study, ethical approval from the ethical committee of the directorate of Primary health care was taken.

This was a cross sectional retrospective study conducted at 12 chronic disease clinics representing the different geographical areas of Al Ahsa starting the month of Dec 2012-March2013. The study population included all the Type 2 diabetic patients who are registered in the Ministry of Health chronic disease clinics situated in Al Ahsa district of Saudi Arabia and are getting medication on regular basis. This population consisted of around 25701 type 2 diabetic cases. The assumption for sample size determination was 67% prevalence of non compliance (as observed by one study in Al Ahsa of Saudi Arabia), a 95% confidence level with a deviation of ±4% from the true prevalence. To calculate representative sample, we used Epi Info (version 6; November, 1993). With the assumption that the complications in the patients with diabetes could be between 65% and 69% and to achieve the confidence level of 95% we needed 532 persons with diabetes. A systematic random sampling was used to select every third diabetic patient from the daily appointment list attending the chronic disease clinic in the selected health centers. The health records of these diabetic patients attending the 12 chronic disease clinics of Al Ahsa region were screened to document the various complications they are suffering from. The data were collected comprising demographic variables (age, sex and marital status), duration of diabetes, BMI and status of diabetic control (Hb1Ac Level) and chronic complications, The level of glycemic control were defined as optimal (HbA1c < 6.5%), fair (6.5% ≤ HbA1c ≤ 7.5%), and poor (HbA1c > 7.5%). The status of obesity was classified according to the international classification based on BMI which were underweight < 18.5, normal 18.5-24.9, overweight 25-29.9, obesity grade I 30-34.9, obesity Grade II35-39.9 and extreme obesity grade III >34.

### 2.1 Complications

Only chronic complications (categorized as cardiovascular conditions, cerebrovascular conditions, nephropathy, ocular lesions, neuropathy, and diabetic foot problems) that developed after the proper diagnosis of T2DM and could be attributed to diabetes were considered in this study. Cardiovascular morbidity included: hypertension, angina, chronic heart failure, myocardial infarction, other related heart diseases, and peripheral vascular disease; the considered cerebrovascular conditions were stroke and transient ischemic attack (TIA); ocular lesions consisted of retinopathy, cataract and blindness; nephropathy included microalbuminuria, macroalbuminuria, renal hypofunction, and renal failure; and diabetic foot problems presented as foot ulcers or amputation (AMP). According to the involved blood vessels, complications were also stratified into macrovascular complications (all cardiovascular, cerebrovascular and foot diseases) and microvascular complications (nephropathies, neuropathy and eye lesions) (Toth et al., 2012).

Only chronic complications (categorized as cardiovascular conditions, cerebrovascular conditions, nephropathy, ocular lesions, and neuropathy including diabetic foot) that developed after the proper diagnosis of Diabetes Mellitus (DM) and could be attributed to diabetes were considered in this study. Cardiovascular complications included: hypertension and Ischemic Heart Disease (IHD); the considered cerebrovascular conditions included stroke and its complication and transient ischemic attack (TIA); ocular lesions included diabetic retinopathy; nephropathy included microalbuminuria, macroalbuminuria, and renal failure; and neuropathy included painful peripheral neuropathy, painless peripheral neuropathy and diabetic foot problem consisted of superficial foot ulcer, deep foot ulcer or amputation (AMP).

Screening criteria for IHD was either patient with typical cardiac symptoms and abnormal resting ECG or asymptomatic patient with abnormal stress tests, either by ECG or echo and nuclear perfusion imaging test. The presence of diabetic nephropathy was based on the presence of microalbuminuria and abnormally high level of serum creatinine level. Those cases that already have renal failure and are on dialysis or getting treatment for renal failure were included as having suffering from diabetic nephropathy. The patients with stroke were those who have been diagnosed as suffering from stroke at the higher center and were admitted for stroke treatment and were suffering from stroke complication. The diabetic cases who complained of an acute episode of temporary neurologic dysfunction that lasted less than an hour and without any associated tissue infarction of the brain tissue were considered as suffering from TIA.

The reports of the ophthalmologist by regular detailed fundus examination were tagged with the patients’ record and were considered for the presence and absence of diabetic retinopathy.

The distal symmetric polyneuropathy screening were performed for each patient of type 2 at diagnosis and yearly afterwards using tests such as pinprick sensation, temperature and vibration perception (Using a 128 Hz tunning fork), 10 gm monofilament pressure sensation at the dorsal surface of both great toes, just proximal to the nail bed and ankle reflexes. The documentation on the patient’s record based on these examinations were included in the data. This consisted of normal foot, neuroischemic foot, foot ulcer or amputed foot.

Serum triglyceride level of 150 mg or less was considered as the optimal, 150-199 mg as borderline, 200-499 mg as high and > 500 mg as very high. Serum cholesterol of 200mg or less was considered as normal while a level of 200-239 mg as borderline and above 240 high as high. Low density lipoprotein C (LDLC) level of 100 or less was considered as the optimal while 100-129 as above optimal, 130-159 as borderline high, 160-189 as high and >190 as very high. A serum High density lipoprotein C (HDLC) level of 40-60 was considered as desirable while >60 as High risk.

### 2.2 Statistical Analysis

A SPSS 16 version was used for all statistical calculations. Results were expressed as mean values ± SD. For nonparametrical distributions the chi square test was used. A p < 0, 05 was considered significant all statistical calculations.

## 3. Results

### 3.1 General Characteristics of Study Population

The medical records of 506 type 2 diabetic subjects could be examined giving a response rate of 95%. Among them were 222 (43.9%) male and 284 (56.1%) female with a mean age of 57.44 years (range 23-88 years). More than fifty percent of them belonged to rural (53%) and the mean duration of diabetes was 10.20 ± 5.96 (range: 1-33 years). Most of the study subjects (89.9%) were married while 5.9% (30) were widow and 4.2% (21) were never married. The mean BMI of the study subjects was 31.43 ± 6.44 (range: 17.8-61.5) [Table 1].

**Table 1:** Demographic and clinical characteristics of the study population

Variables	Percentage	Number
**Gender**		
Male	43.9	222
Female	56.1	284
**Geographic distribution**		
Rural	53	268
Urban	47	238
**Marital Status**		
Married	89.9	455
Never married	4.2	21
Widow	5.1	30
**Mean age of the participant**	57.44 years (range 23-88 years)	
**Mean duration of diabetes**	10.20 ±5.96 (range: 1-33 years)	
**Mean BMI**	31.43 ± 6.44 (range: 17.8-61.5)	
**Diabetic control**		
Optimal	11.1	56
Fair	17.6	89
Poor	71.3	361
**Co morbidity Blood Pressure**		
Normal	52.2	264
Hypertension	47.8	242
**BMI**		
Normal weight	10.1	51
Overweight	35.8	181
Obese Class1	30.0	152
Class2	12.8	65
Class3	11.3	57
**Serum Triglyceride**		
Normal	57.5	291
Borderline	18	95
High	24.1	118
Missing	0.4	2
**Serum Cholesterol**		
Normal	60.6	307
Borderline High	27.3	138
High	11.7	59
Missing	0.4	2
**Serum LDLc**		
Optimal	13.8	70
Missing	30.5	154
**Serum HDLc**		
High	55.7	282
Above optimal level	61.3	310
Missing	38.7	196

Only 11.1% of the diabetic patients were optimally controlled while 17.6% were fairly controlled and 71.3% were poorly controlled. More than fifty percent subjects were obese (30% with class 1, 12.8% with Class 2 and 11.3% with Class 3), 35.8% were overweight and only 10.1% were of normal weight. Serum triglyceride level of 57.9% of the subjects was under normal range while 18% and 24.1% of the subjects were having borderline high and high level of triglyceride respectively. Serum cholesterol level of 60.6% of diabetic patients was optimal while that of 27.3% and 11.7% were at borderline and high level respectively.

The serum LDLc of about 61% (308) of the subjects could be found on their health record which showed that 36.25 % of these subjects were under the optimal level while 68.75% of them showed either high or very high level. Likewise the serum HDLC of 38.7% of the study subjects was not found on the health records. However the HDLC level of all these subjects were under desirable level [Table 1].

### 3.2 Prevalence of Chronic Complications

Overall 72.72% (95% CI 69.78-74.45) of the study subjects were suffering from one or more complications of diabetic mellitus. With regard to the prevalence of chronic complications of Type 2 DM across categories (organ or system), 32.8% (165) of the 506 subjects had single-category complications while there were 25.29% (128) with two and 15% (75) with more than two complications respectively. The single category complication consisted of cardiovascular, renal, diabetic retinopathy, cerebrovascular, diabetic foot and peripheral neuropathy; the prevalence of which were 22.72%, 4.15%, 2.96%, 0.19 %, 2.17 % and 1.38% respectively [Table 2].

**Table 2:** Prevalence of chronic complications of Type 2 DM across categories (organ or system)

Variables	No of subjects	%
No Complication	138	27.28%
Chronic Complication	368	72.72%
**Categories by organ and system**		
Cardiovascular	115	22.72
Hypertension	99	19.56
IHD	10	1.98
Post CBAG	2	0.3%
Heart failure	3	0.5
Renal	21	4.15
Microalbiminuria	18	3.56
Renal failure	3	0.6
Ocular	15	2.96
Early non proliferative diabetic retinopathy	6	1.18
Moderate Non proliferative diabetic retinopathy	7	1.38
Proliferative diabetic retinopathy	2	0.59
Cerebrovascular	1	0.19
Stroke	1	0.19
Diabetic foot	11	2.17
Superficial foot ulcer	7	1.38
Deep foot ulcer	2	0.39
Foot amputation	2	0.39
Peripheral Neuropathy	7	1.38
Painless sensory neuropathy	4	0.79
Painful sensory neropathy	3	0.59
Single category	169	33.39
Two category	128	25.29
Three or more	75	15.0

The overall prevalence of hypertension, cardiovascular, renal, ocular, cerebrovascular, diabetic foot and peripheral neuropathy were 52.2%, 26.48%, 23.8%, 19.7%, 1.8%, 12.9% and 15.2% respectively. The cardiovascular complication consisted of IHD (118, 23.3%), heart failure (10, 2.0%) and cases with past CBAG surgery (6, 1.2%). The renal complication consisted of 19.6% (99) with microalbiminuria and 4% (20) with renal failure. The ocular complication consisted of early non proliferative diabetic retinopathy, moderate non proliferative diabetic retinopathy and proliferative diabetic retinopathy, the prevalence of which were 13.0% (66), 5.3% (27) and 1.4% (7) respectively. The total subjects with cerebrovascular complication consisted of 1% (5) with TIA and 0.8% (4) with history of stroke. The diabetic foot consisted of 10.9% (55) with superficial foot ulcer, 1.2% (6) with deep foot ulcer and 0.8% (4) with foot amputation. The peripheral neuropathy consisted of 9.6% (48) suffering from symmetric painless sensory neuropathy and 5.7% (29) suffering from painful peripheral neuropathy [Table 3].

**Table 3:** The overall complication of type 2 diabetics

Complications	No.	%
**Cardio vascular**		
IHD	118	23.32
Chronic heart failure	10	1.97
Post CBAG	6	1.85
Total	134	26.48
Hypertension	264	55.2
**Renal**		
Microalbiminuria	99	19.8
Renal failure	20	4.0
Total	119	23.8
**Ocular**		
Early non proliferative diabetic retinopathy	56	13
Moderate non proliferative diabetic retinopathy	27	5.3
Proliferative diabetic retinopathy	7	1.4
Total	90	19.7
**Cerebrovascular**		
TIA	5	1.0
Stroke	4	0.8
Total	9	1.8
**Diabetic foot**		
Superficial foot ulcer	54	10.7
Deep foot ulcer	7	1.4
Foot amputation	4	0.8
Total	65	12.9
**Peripheral neuropathy**		
Painless peripheral neuropathy	48	9.5
Painful peripheral neuropathy	29	5.7
Total	77	15.2

### 3.3 Demographic and Geographic Stratification of Chronic Complications

The overall prevalence of complication among the female subjects was significantly higher than the male (78.16%, 95% CI 76.76-84.40 Vs 65.76%, 95% CI 61.63-69.89, p=.038). This was also true with the single (35.21, 95% CI 31.17-39.49 Vs 29.28%, 95% CI 25.31-3.23, p=.039) double (25.70%, 95% CI 23.32-31.08 Vs 22.97%, 95% CI 19.3-26.6, p=.039) and multiple complications (17.25% 95% CI 12.38 18.7 Vs 13.51%, 95% CI 10.53 -16.49, p=.039) where the prevalence was significantly higher among the females than the males. The prevalence of chronic complication of type 2 diabetes mellitus was higher among the urban population than the rural population (77.3% 95% CI 72.88-80.26 Vs 69.78% 95% CI 66.1%-76.92%, p=.035). Both the single and double complications were significantly more common among the urban while the multiple complications were significantly higher among the rural population than the urban population (16.79%, 95% CI 13.59- 20.11 Vs 12.18%, 95% CI 10 - 12.23) [Table 4].

**Table 4:** Demographic and Geographic stratification of chronic complications

	Gender			Geographic region		

	Male	Female	P value	Urban	Rural	P value
No complication	76(34.23)	62(21.83)		54 (22.69)	81 (30.22)	
Categories of Complication						
Single complication	65 (29.28)	100(35.21)	.039	89 (37.39)	80 (29.85)	.035
Two complications	51 (22.97)	73 (25.70)	.038	66 (27.73)	62(23.14)	000
Three and more complications	30 (13.51)	49 (17.25)	.039	29(12.18)	45 (16.79)	000
Total	146(65.76)	222 (78.16)	.038	184(77.3)	187 (69.78)	.035

### 3.4 Gender Difference of Co-Morbidity Factors

Hyperlipidemia was more prevalent among the female diabetics (45.42 Vs 38.1, p=.295) but the difference was not statistically significant. More than ninety percent of the female were either overweight or obese which was significantly more than the male subjects (91.13%, 95% CI 88.68-93.58 Vs 86.84, 95% CI 83.92-89.76, p=.000). The same was true with the serum cholesterol level which was significantly higher among the female than their male counterpart (40.27, 95% CI 36.04-44.50 Vs 29.58, 95% CI 25.64-33.52, p=.007) [Table 5].

**Table 5:** Gender difference of co-morbidity factors

Comorbidity variables	Male, n=222 N(%)	Female, n=284 N(%)	Total n =506 N(%)	P value
**Serum Triglyceride**				.295
Normal	136(62.26)	155(54.58)	291 (57.51)	
Borderline high	39(17.57)	52(18.31)	91(17.99)	
High	43(19.37)	75(26.41)	118(23.32)	
Very High	4(1.8)	2(0.7)	6(1.11)	
**Blood Pressure**				.004
Normal	143(67.14)	194 (66.22)	337 (66.61)	
Hypertension	70(32.86)	99(33.78)	169(33.39)	
**Obesity**				.000
Normal weight	28(12.62)	23(8.1)	51(10.08)	
Overweight	92(41.45)	89(31.34)	181(35.78)	
Obese Class 1	73(32.88)	79(27.82)	152 (30.04)	
Obese Class2	15(6.75)	50(20.83)	65(12.84)	
Obese Class 3	14(6.3)	43(17.60)	57(11.26)	
**Serum Cholesterol**				.000
Normal	156(70.27)	151(53.17)	307(60.67)	
Borderline high	44(19.82)	96(33.80)	140(27.67)	
High	22 (9.91)	37(13.03)	59(11.66)	

## 4. Discussion

The diabetes mellitus is well known for its significant effect on the morbidity and mortality of the population caused by its micro and macro vascular complications resulting in huge burden on the health care system. The knowledge of the epidemiology of its Co-morbidity factors and the prevalence of its complication is very important for formulating the necessary policies and action plan. In the recent years, there have been only a handful small scale studies dealing with the prevalence of chronic complications among diabetes patients attending the Primary health Care facilities of Ministry Of Health in Saudi Arabia. The present study was conducted in the Al Ahsa district of Saudi Arabia and consisted of the entire registered diabetic patient attending all the Primary health care facilities of the ministry of health.

This study has shown that 72.72% of the type 2 diabetic patients were suffering from one or more chronic complication which is lower than that observed in Malaysia (78%) (Abougalambou et al., 2011) but higher than what observed in Libya (68.7%) (Roaeid & Kadiki, 2011) India (60%) (Vaz, Ferreira, Kulkarni, Vaz, & Pinto, 2011), China (Liu, Fu, Wang, & Xu, 2010) (52%) and Iran (51.9%) (Afkhami-Ardekani & Zahmatkash, 2009). However these differences may be due to different methodology and different epidemiological environment.

Unlike our study, Pakistani study (Shafiqur-Rahman, 2004) has found lower percentage of diabetic patients with single complication (17% with single, 33% with two and 35% with three complications). However in Libyan study (Roaeid & Kadiki, 2011), 36.7% had one, 20.1% had two and 11.9% had three and more complication and the same was true with Chinese study (Liu et al., 2010) where 30.5% subjects had single category complication while 15.4% and 6.2 % were suffering from three or more complications respectively. This difference might be due to associated co morbidity and the different duration of diabetes among the diabetic population.

Many studies have found remarkable rural urban differences in the prevalence of type 2 diabetes and so also in its chronic complications (Balasuriya, Sumanatilleke, Jayasekera, Wijesuriya, & Somasundaram, 2012; Zimmet et al., 1981). The prevalence of Type 2 diabetes in the urban population has been established higher than the rural population and so its complications are also expected to be higher in urban population. With this expectation we have found a higher prevalence of chronic complications (76.57% Vs 72.01, p=.185) among the urban diabetic population than the rural but the same was true in a study conducted in Uzbekistan (Ismailov, Berdykulova, & Khaidarova, 2004) where the researchers have found a higher prevalence of late complications of diabetes in urban population (p<0.001).

A very high prevalence of obese diabetic (n=274, 54.1%), poorly controlled diabetics (n=361, 71.3%), a higher level of lipidaemia (n=215, 42.9%) and cholesterol (n=199, 39.4%), and a significantly higher prevalence of hypertension (n=264, 52.2%) among the diabetic patients in our study is a matter of concern as they are strong co morbidity factors for further complication.

LDLc and HDLc are the known risk factors for the micro vascular complication especially among the diabetics (Toth et al., 2012). Routine measurement of LDLc and HDLc is required for all the diabetic patients. In this study we have found that the serum LDLc and HDLc level of 39% (197 of 506) of patients were not documented on the health record of the patients and so one of the important risk factors for micro vascular complication is largely unknown.

## 5. Conclusion

We have found a high percentage of chronic complications among the diabetic patients. With the reported high percentage of obesity, hypertension and dyslipidaemia among them as the co morbidity factors which if not controlled can cause an increased number of chronic complication. A high percentage of poorly controlled diabetic subjects is a matter of concern. Lacking lipid profile of the diabetic patients in our study require special attention on the timely investigations of this very important risk factors.

## References

[ref1] Abougalambou S. S. I, Hassali M. A, Sulaiman S. A. S, Abougalambou A. S (2011). Prevalence of Vascular Complications among Type 2 Diabetes, Mellitus Outpatients at Teaching Hospital in Malaysia. J Diabetes Metab.

[ref2] Afkhami-Ardekani M, Zahmatkash M (2009). Prevalence of Type 2 Diabetes Complications and their Contributing Factors in Yazd Province. Iranian Journal of Diabetes & Obesity (IJDO).

[ref3] Al-Homrany M. A, Abdelmoneim I (2004). Significance of proteinuria in type 2 diabetic patients treated at a primary health care center in Abha City, Saudi Arabia. West Afr J Med.

[ref4] Al-Maskari F, El-Sadig M, Norman J. N (2007). The prevalence of macrovascular complications among diabetic patients in the United Arab Emirates. Cardiovascular diabetology.

[ref5] Alwakeel J. S, Al-Suwaida A, Isnani A. C, Al-Harbi A, Alam A (2009). Concomitant macro and microvascular complications in diabetic nephropathy. Saudi journal of kidney diseases and transplantation.

[ref6] Balasuriya B. M. A. C, Sumanatilleke M. R, Jayasekera T. I, Wijesuriya M. A, Somasundaram N. P (2012). Prevalence of micro and macrovascular complications of diabetes detected at single visit screening. Sri Lanka Journal of Diabetes Endocrinology and Metabolism.

[ref7] Caring for Diabetes.com (2004). Epidemiology and Disease Pathology of Diabetic Nephropathy. http://www.caringfordiabetes.com/Epidemiology-andPathology/MicroComplications/EDP_Diabetic_Nephropathy.cfm.

[ref8] Currie C. J, Morgan C. L, Gill L, Stott N. C. H, Peters J. R (1997). Epidemiology and Costs of Acute Hospital Care for Cerebrovascular Disease in Diabetic and Nondiabetic Populations. Stroke.

[ref9] Farag Y. M, Al Wakeel J. S (2011). Diabetic Nephropathy in the Arab Gulf Countries. Nephron clinical practice.

[ref10] Ismailov S. I, Berdykulova D. M, Khaidarova F. A (2004). Late Complications of Diabetes mellitus in the Republic of Uzbekistan. Heart Drug.

[ref11] Kengne A. P, Mbanya J. C (2005). Heart Disease in Africa. AMA.

[ref12] Khan A. R, Wiseberg J. A, Lateef Z. A, Khan S. A (2010). Prevalence and determinants of diabetic retinopathy in Al Hasa region of Saudi Arabia: primary health care centre based cross-sectional survey 2007-2009. Middle East Afr J Ophthalmol.

[ref13] Liu Z, Fu C, Wang W, Xu B (2010). Prevalence of chronic complications of type 2 diabetes mellitus in outpatients - a cross-sectional hospital based survey in urban China. Health and Quality of Life Outcomes.

[ref14] Roaeid R. B, Kadiki O. A (2011). Prevalence of long-term complications among Type 2 diabetic patients in Benghazi. Libya Journal of Diabetology.

[ref15] Shafiqur-Rahman I. Z (2004). Prevalence of microvascular complications among diabetic patients. Pakistan J. Med. Res.

[ref16] Sierra G. N (2009). The global pandemic of diabetes. AJDM.

[ref17] Toth P. P, Simko R. J, Palli S. R, Koselleck D, Quimbo R. A, Cziraky M. J (2012). The impact of serum lipids on risk for microangiopathy in patients with type 2 diabetes mellitus. Cardiovascular diabetology.

[ref18] Vaz N. C, Ferreira A. M, Kulkarni M. S, Vaz F. S, Pinto N. R (2011). Prevalence of diabetic complications in rural Goa, India. Indian J Community Med.

[ref19] Zimmet P, Faaiuso S, Ainuu J, Whitehouse S, Milne B, de Boer W (1981). Theprevalence of diabetes in the rural and urban Polynesian population of Western Samoa. Diabetes.

